# Effect of Small Retrofitting Changes to Daily Living Environments for Supporting the Bodies of Frail Older Adults

**DOI:** 10.1093/geroni/igaf122.2625

**Published:** 2025-12-31

**Authors:** Satoru Handa, Ayano Nomura, Shihomi Kuribayashi, Koji Kitamura, Hisashi Kawai, Yoshifumi Nishida

**Affiliations:** Institute of Science Tokyo School of Engineering, Tokyo, Tokyo, Japan; Institute of Science Tokyo School of Engineering, Tokyo, Tokyo, Japan; Institute of Science Tokyo School of Engineering, Tokyo, Tokyo, Japan; National Institute of Advanced Industrial Science and Technology, Kashiwa, Chiba, Japan; Tokyo Metropolitan Institute for Geriatrics and Gerontology, Itabashi City, Tokyo, Japan; Institute of Science Tokyo School of Engineering, Tokyo, Tokyo, Japan

## Abstract

As the aging population continues to grow, it is essential to support frail older adults in leading safer and more independent daily lives. One promising approach is to change environmental design to improve physical stability. In the previous study, we examined the effects of attaching handrails and finger-grip protrusions to desks. Our findings suggested that these design modifications could contribute to an improved standing posture. In this study, we focused on the effect of additive small changes, such as modifying the desk’s surface material and adding edge protrusions of 3 mm, 5 mm, and 7 mm, to present the possibility of practical solutions in real-life settings. We conducted experiments with 59 older adults (aged 65–98), including senior car users, measuring their standing motion using the modified desks. The results were analyzed through cluster analyses, comparing desks with conventional wooden surfaces to those with alternative materials. Our findings revealed that desks with rubberized resin or polyvinyl surfaces allowed for greater forward force application compared to standard wooden desks. Additionally, when edge protrusions of 3 mm, 5 mm, or 7 mm were introduced, the cluster analyses demonstrated improved physical stability compared to desks without protrusions. Notably, some older adults were able to exert a forward force equivalent to 0.1 times their body weight when using a desk with a 7 mm edge protrusion. These results suggest that even small design modifications that can be retrofitted can effectively enhance physical stability during movement for some older adults.

